# Negative Regulation of mTOR Signaling Restricts Cell Proliferation in the Floor Plate

**DOI:** 10.3389/fnins.2019.01022

**Published:** 2019-09-25

**Authors:** Minori Kadoya, Noriaki Sasai

**Affiliations:** Developmental Biomedical Science, Graduate School of Biological Sciences, Nara Institute of Science and Technology, Ikoma, Japan

**Keywords:** chick, neural tube, floor plate, mTOR, cell proliferation

## Abstract

The neural tube is composed of a number of neural progenitors and postmitotic neurons distributed in a quantitatively and spatially precise manner. The floor plate, located in the ventral-most region of the neural tube, has a lot of unique characteristics, including a low cell proliferation rate. The mechanisms by which this region-specific proliferation rate is regulated remain elusive. Here we show that the activity of the mTOR signaling pathway, which regulates the proliferation of the neural progenitor cells, is significantly lower in the floor plate than in other domains of the embryonic neural tube. We identified the forkhead-type transcription factor FoxA2 as a negative regulator of mTOR signaling in the floor plate, and showed that FoxA2 transcriptionally induces the expression of the E3 ubiquitin ligase RNF152, which together with its substrate RagA, regulates cell proliferation via the mTOR pathway. Silencing of RNF152 led to the aberrant upregulation of the mTOR signal and aberrant cell division in the floor plate. Taken together, the present findings suggest that floor plate cell number is controlled by the negative regulation of mTOR signaling through the activity of FoxA2 and its downstream effector RNF152.

## Introduction

The neural tube is the embryonic precursor to the central nervous system, and is composed of neural progenitor cells and postmitotic neurons (Ribes and Briscoe, 2009; Le Dreau and Marti, 2012). These cells are arranged in a quantitatively and spatially precise manner, which ensures that the neural tube develops into a functional organ.

The growth of the neural tube depends on the balance between progenitor cell proliferation and neuronal differentiation (Kicheva et al., 2014), and accumulating studies have revealed the molecular mechanisms underlying this coordination. For instance, Notch signaling maintains the progenitor state to allow self-renewal (Shimojo et al., 2011; Molina and Pituello, 2017). Hippo signaling, which is mediated by Tead/Yap transcription factors, is involved in progenitor cell proliferation by inducing cyclin D1 expression (Cao et al., 2008; Molina and Pituello, 2017). On the other hand, a group of basic helix-loop-helix (bHLH) transcription factors known as proneural genes promote neuronal differentiation (Shimojo et al., 2011; Molina and Pituello, 2017; Baker and Brown, 2018).

In addition to these programs by which proliferation and differentiation are regulated, each neural progenitor cell has its own character. In the trunk neural tube, the progenitor cells are divided into more than ten subtypes, which include the roof plate, pD1-pD6, p0-p2, pMN, p3, and the floor plate (FP) domains arrayed in this order from dorsal to ventral (Ribes et al., 2010; Alaynick et al., 2011), and most of these cells further differentiate into their corresponding neurons. The distribution of the cells, or the pattern formation of the neural tube, is regulated by secreted factors, collectively called morphogens (Dessaud et al., 2008; Perrimon et al., 2012; Kicheva et al., 2014). Wnt, BMP, and Sonic Hedgehog (Shh) are representative morphogens, and each neural domain is thought to be assigned according to morphogen concentrations (Jacob and Briscoe, 2003). Importantly, each domain has its own size; some domains have a large number of cells, whereas the others have only a few (Kicheva et al., 2014). The determination of cell number in each neural domain is achieved by the combinatorial control of the cell identities by morphogenic signals, cell proliferation and timing of the differentiation.

Shh is expressed in the FP and its underlying mesodermal tissue notochord, and the protein is distributed in a gradient from the ventral to the dorsal regions, with the highest level in the FP (Ribes and Briscoe, 2009). Shh plays important roles in the assignment of ventral neural identity (Jacob and Briscoe, 2003; Dessaud et al., 2008).

Shh regulates cell proliferation in parallel with the cell specification (Komada, 2012). Embryos devoid of the *Shh* gene exhibit not only defective pattern formation, but also a reduced size of the neural tube, suggesting that Shh plays indispensable roles in both cell proliferation and tissue growth (Chiang et al., 1996; Bulgakov et al., 2004). Conversely, sustained and excessive Shh signaling leads to tumorigenesis (Rowitch et al., 1999; Dahmane et al., 2001). The Shh signal, therefore, needs to be strictly regulated both spatially and temporally.

The floor plate, located at the ventral-most part of the neural tube, is a source of Shh, and acts as an organizer for D-V pattern formation of the neural tube (Dessaud et al., 2010; Yu et al., 2013). In addition, the FP has a number of unique characteristics compared with other neural domains (Placzek and Briscoe, 2005). At the trunk level, the FP is non-neurogenic (Ono et al., 2007), which is distinct from other progenitor domains where these cells differentiate into the corresponding neurons (Dessaud et al., 2008; Ribes and Briscoe, 2009). FP cells express guidance molecules such as Netrin and Slit, which are essential for the precise guidance of the commissural axons (Kennedy et al., 1994; de la Torre et al., 1997; Ming et al., 1997; Sloan et al., 2015). The FP also expresses actin-related factors, and is important for defining the neural tube shape (Nishimura and Takeichi, 2008; Nishimura et al., 2012). Therefore, the FP is indispensable for pattern formation, morphology, and functional control of the entire neural tube.

Neural progenitor cells in any neural domain dynamically increase in number, whereas the FP cells, which are exposed to the highest level of Shh, show significantly low levels of proliferation (Kicheva et al., 2014). One possible explanation for this phenomenon is the presence of a negative regulator(s) of cell proliferation that is exclusively expressed in the FP region and antagonizes the proliferative effect of Shh.

The mechanistic target of rapamycin (mTOR) pathway is a versatile signaling system involved in a number of biological events including cell proliferation, survival and metabolism through early embryonic to postnatal stages (Gangloff et al., 2004; Murakami et al., 2004; Laplante and Sabatini, 2009). The mTOR complex is the hub of mTOR signaling (Laplante and Sabatini, 2012), and acts as a serine/threonine kinase. Unsurprisingly, mTOR signal is essential for proper development of the central nervous system (Tee et al., 2016; Ryskalin et al., 2017; LiCausi and Hartman, 2018), and aberrant mTOR signaling causes neural defects during development. For instance, blocking the mTOR signal with the phosphoinositide 3-kinase and mTOR inhibitors represses neurogenesis (Fishwick et al., 2010). Genetic elimination of the mTOR signal disrupts progenitor self-renewal and brain morphogenesis (Ka et al., 2014). Tuberous sclerosis complex subunit 1 (Tsc1) is a negative regulator of mTOR signaling (Dalle Pezze et al., 2012), and *Tsc1* homozygous mutant mice exhibit embryonic lethality with an unclosed neural tube (Rennebeck et al., 1998; Kobayashi et al., 2001).

Previous studies revealed associations between the mTOR and Shh signaling pathways in the neural tube. First, the mTOR pathway is active in ventral regions and in migrating neural crest cells, as shown by the expression of the phosphorylated form of mTOR (Nie et al., 2018). Because Shh is important for the assignment of ventral neural domains (Ribes and Briscoe, 2009) and migration of the neural crest (Kahane et al., 2013), the distribution of activated mTOR suggests that the Shh and the mTOR signaling pathways act in cooperation with each other.

It has also been shown that the mTOR pathway phosphorylates and activates the transcription factor Gli1, a mediator of intracellular Shh signaling, and promotes the expression of target genes related to cell proliferation (Wang et al., 2012), thus supporting the relationship between mTOR and Shh signaling. Gli1 activation by mTOR is recognized as non-canonical in terms of the Gli activation, as this pathway is independent from the one mediated by the receptor protein for the Shh signal, Smoothened (Smo) (Dessaud et al., 2008). However, this signaling pathway was demonstrated at the cellular level, and whether this pathway is also functional in a developmental context remains elusive.

A recent study showed that abrogation of cilia activates the mTOR signal (Foerster et al., 2017). As Shh signaling requires cilia (Sasai and Briscoe, 2012), this finding suggests that Shh signaling, and mTOR activation are somehow related to each other. However, the detailed mechanisms remain to be elucidated (Foerster et al., 2017).

In the present study, we mainly used chick embryos to investigate the mechanisms underlying the selective low proliferation rate of FP cells, with particular focus on the relationship between the Shh and mTOR signaling pathways. FoxA2, a transcription factor expressed in the FP and a target gene of Shh, blocked the mTOR signal, thereby altering cell proliferation. We identified the E3 ubiquitin ligase RNF152 as a target gene of FoxA2, and showed that RNF152 negatively regulates mTOR signaling by catalyzing the ubiquitination of the small GTPase RagA. Loss-of-function experiments were performed to examine the role of RNF152 in regulating the proliferation of FP cells.

## Results

### The FP Is Significantly Less Proliferative Than Other Neural Domains

**FIGURE 1:**
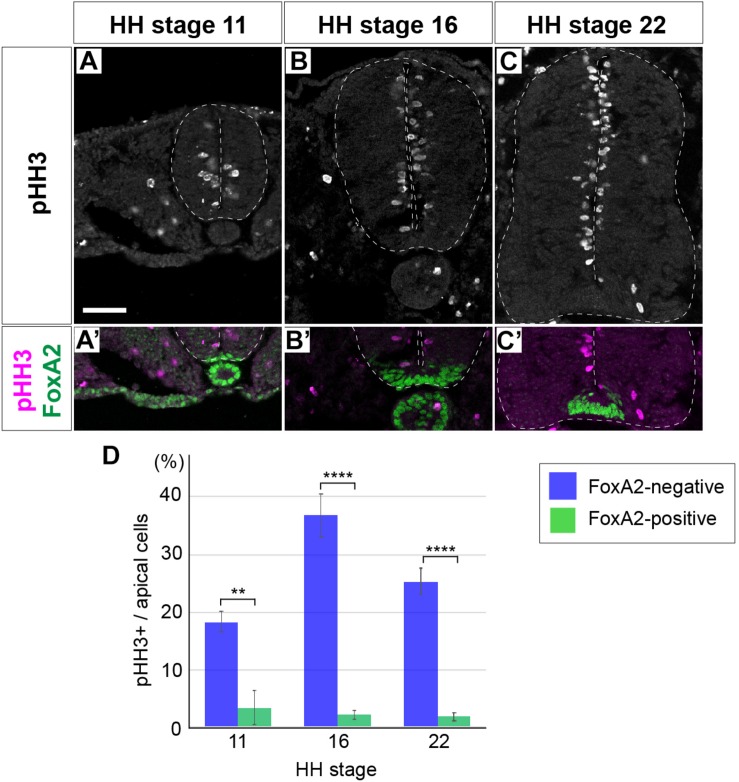
Floor plate cells are not proliferative unlike the neural progenitor cells of other domains. **(A–C’)** Expression of pHH3 and FoxA2 in neural tube sections at HH stage 11 **(A,A’)**, 16 **(B,B’)**, and 22 **(C,C’)**. pHH3-positive cells were not detected in the FP, where FoxA2 is highly expressed **(A’,B’,C’)**. **(D)** Quantitative data of the ratio of pHH3-positive cells over the apical cells in the FP and in other domains of the neural tube. Scale bar = 50 μm. ∗∗*p* < 0.01, ∗∗∗∗*p* < 0.0001.

To clarify the mechanisms underlying the regulation of cell proliferation and tissue growth of the neural tube, the distributionof mitotic cells was examined by immunofluorescent detection of the mitotic marker phospho-Histone 3 (Ser 10) (pHH3)-positive cells in cross sections of the neural tube. Embryos were harvested at Hamburger and Hamilton (HH) stage 11, soon after neural tube closure, HH stage 16, at the start of neurogenesis, and HH stage 22, when the neural tube matures; and pHH3 expression was analyzed at the anterior thoracic level. pHH3-positive cells were detected in the apical region of the neural tube (Figures 1–C).

As reported before, whereas pHH3-positive cells were found almost uniformly throughout the D-V axis, only a very few positive cells were found in the FP (Kicheva et al., 2014). We hypothesized the existence of a negative regulator for the cell proliferation in the FP. One such candidate was the forkhead-type of a transcription factor FoxA2 (Sasaki and Hogan, 1994), because FoxA2 is expressed with a high level in the FP (Figures 1A’,B’,C’; Sasai et al., 2014). Therefore, in order to find the correlation between FoxA2 and pHH3, we counted the pHH3 cells in the FoxA2-positive and -negative apical cells. The results showed that pHH3-positive cells were actually rarer in the FP, or in the FoxA2-positive area, than in the other region of the neural tube at any stage (Figures 1A’,B’,C’,D).

Taken together, the cell proliferation activity was significantly lower in the FoxA2-expressing FP cells in the neural tube.

### mTOR Signal Is Inactive in the FP

We next explored the mechanisms underlying the regulation of cell proliferation in the neural tube. As the mTOR signaling pathway is important for cell proliferation in many biological contexts (Saxton and Sabatini, 2017), we became interested in the possible involvement of mTOR signaling in the FP development.

**FIGURE 2:**
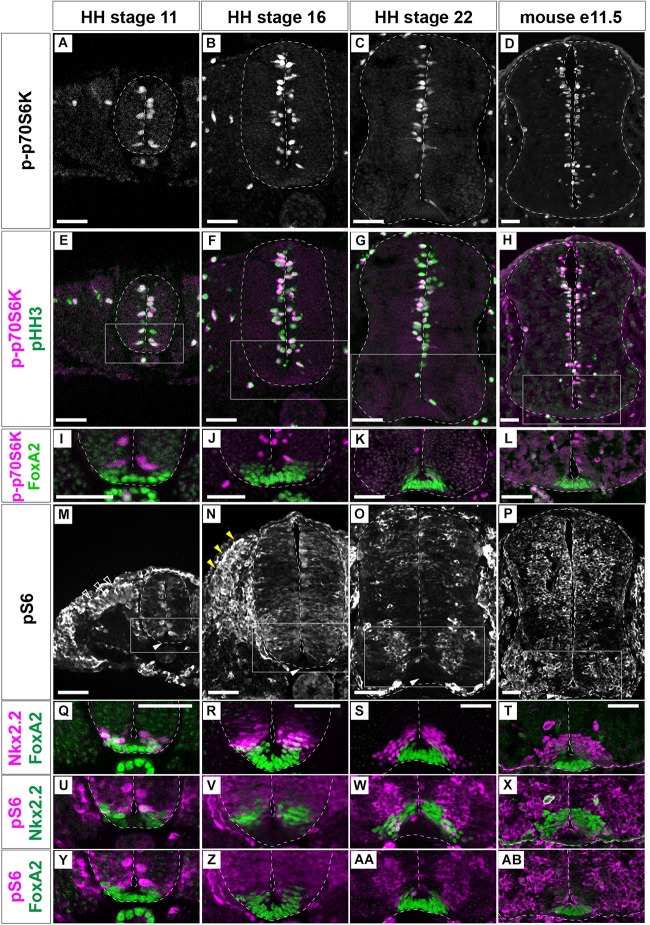
mTOR signal is negative in the floor plate. p-p70S6K **(A–L)**, pHH3 **(E–H)**, and FoxA2 **(I–L)** expression was identified by immunofluorescence at HH stages 11 **(A,E,I)**, 16 **(B,F,J)** and 22 **(C,G,K)** of chick, and at e11.5 **(D,H,L)** mouse neural tube sections. **(M–AB)** pS6-positive cells (white, **M–P**; and magenta, **U–AB**) were analyzed with those of Nkx2.2 (magenta, **Q–T**; green, **U–X**) and FoxA2 (green, **Q–T,Y–AB**). **(I–L)** correspond to the areas surrounded by rectangles in **(E–H)**, respectively. **(Q,U,Y)**,**(R,V,Z)**,**(S,W,AA)**, and **(T,X,AB)** correspond to the areas surrounded by rectangles in **(M–P)**, respectively. Scale bars = 50 μm. White arrowheads in **(M–P)**; outlined arrowheads in **(M)**; dorsal somite, yellow arrowheads in **(N)**; dermomyotome.

The distribution of cells active for the mTOR signal along the D-V axis of the neural tube was analyzed. For this purpose, we evaluated two major readouts of mTOR activity, phospho-p70S6K (p-p70S6K) and phosphorylated-S6 ribosomal protein (Ser235/236) (hereafter pS6), which can be phosphorylated by p70S6K, by immunofluorescence, at the anterior thoracic level of chick neural tube (Figures 2A–C,E–G,I–K,M–O,Q–S,U–W, Y–AA) and of the mouse embryos (Figures 2D,H,L,P,T,X,AB; Biever et al., 2015).

p-p70S6K-positive cells were distributed at the apical region of the neural tube at any stages of chick (Figures 2A–C) and mouse (Figure 2D) neural tube. Moreover, importantly, p-p70S6K-positive cells were also positive for pHH3 (Figures 2E–H), suggesting that the mTOR signal is involved in cell proliferation.

pS6 was detected at the apical domain of the neural tube at HH stage 11 (Figure 2M; Fishwick et al., 2010). At HH stage 16, pS6 was found almost throughout the neural tube with variations in signal intensity (Figure 2N). At HH stage 22, pS6 was detected at the transition zone between progenitor and postmitotic neurons (Figure 2O). On the other hand, in e11.5 mouse neural tube, a strong pS6 signal was detented in the progenitor regions (Figure 2P), suggesting a species-specific distribution of pS6.

While S6 is a substrate of p70S6K, p-p70S6K-, and pS6-positive areas did not completely coincide. This is because the different subcellular localization of two proteins; p-p70S6K is apparently localized at the nucleus, while pS6 is localized at the cytoplasm. In addition, pS6 can also be phosphorylated by kinases other than p70S6K (Biever et al., 2015).

Although mTOR signaling activation, as detected by p-p70S6K and pS6 expression, was dynamic, neither p-p70S6K nor pS6 were detected in the ventral-most domain at any stage (Figures 2I–L,Y–AB). To more precisely identify pS6-positive cells, pS6-positive domains were compared with FoxA2- and Nkx2.2-expressing domains (Ribes et al., 2010; Sasai et al., 2014). This analysis was performed considering that FoxA2 is weakly expressed in part of the Nkx2.2-positive p3 domain (Figures 2Q–T), and the *bona fide* FP is defined by FoxA2-positive and Nkx2.2-negative regions (Ribes et al., 2010; Sasai et al., 2014). The results showed that the ventral end of pS6 expression coincided with the p3 domain, suggesting that the mTOR signal is active in almost all domains in the progenitor regions of the neural tube, but not in the FP (Figures 2U–AB).

Taken together, these results indicate that mTOR signaling is active in a region-specific manner, and is particularly inactive in FP cells, during neural tube development.

### FoxA2 Blocks Cell Proliferation by Negatively Regulating the mTOR Signal

**FIGURE 3:**
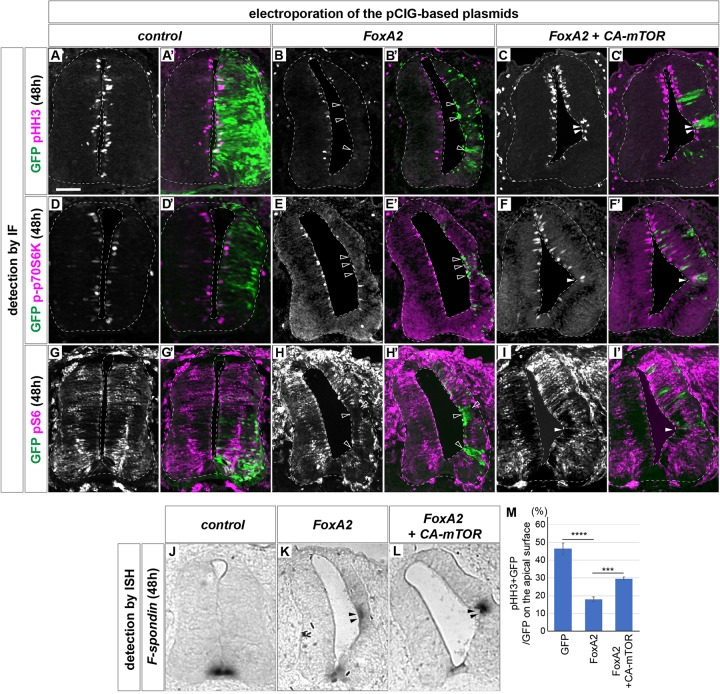
FoxA2 negatively regulates the cell proliferation by blocking the mTOR signal. FoxA2 blocks phosphorylation of p70S6K and S6, and proliferation of the cells without inducing programmed cell death. Plasmids expressing control GFP **(A,A’,D,D’,G,G’,J)**, FoxA2 **(B,B’,E,E’,H,H’,K)** or FoxA2 together with CA-mTOR **(C,C’,F,F’,I,I’,L)** were electroporated into one side of the neural tube of HH stage 12 embryos and the phenotypes were analyzed at 48 hpt by immunofluorescence with pHH3 **(A–C’)**, p-p70S6K **(D–F’)**, pS6 **(G–I’)**, and GFP **(A’–L’)** antibodies. The merged cells of pHH3 **(C,C’)**, p-70S6K **(F,F’)**, or pS6 **(I,I’)** with GFP expression are indicated by filled arrowheads, and the pHH3- **(B,B’)**, p-p70S6K- **(E,E’)**, and pS6- **(H,H’)** negative on GFP-positive cells are indicated by outlined arrowheads. **(J–L)** The cell fate determination for FP by FoxA2 is not altered by CA-mTOR. *F-spondin*-positive cells were identified by *in situ* hybridization. The *F-spondin* expression ectopically induced by FoxA2 is indicated by filled arrowheads **(K,L)**. Analysis in **(J–L)** were performed on the adjacent sections of **(G–I)**, respectively. **(M)** Quantitative data for **(A–C’)**. Scale bar = 50 μm. ∗∗∗*p* < 0.001, ∗∗∗∗*p* < 0.0001.

We next focused on the function of FoxA2 (Ang et al., 1993; Sasaki and Hogan, 1994). FoxA2 is one of the primary responsive genes of Shh (Vokes et al., 2007; Kutejova et al., 2016) and is essential for FP differentiation (Sasaki and Hogan, 1994; Placzek and Briscoe, 2005). Because there are few pHH3-positive cells in the FoxA2-expressing area (Figures 1A’,B’,C’,D), we reasoned that the low proliferation of the FP is controlled by FoxA2. To address this hypothesis, FoxA2 was overexpressed at HH stage 11 on one side of the neural tube, and the phenotypes were analyzed at 48 hpt. We found the FoxA2-overexpressing side was significantly smaller (*n* = 8/8) than the control side (*n* = 0/6) (Figures 3A–B’). Consistently, the number of pHH3-positive cells was significantly lower in FoxA2-overexpressing cells (*n* = 8) than in the control GFP-expressing neural tube (*n* = 6), suggesting that FoxA2 blocks cell cycle progression (Figures 3A–B’,M). There were fewer cells positive for p-p70S6K (*n* = 8/8; Figures 3D–E’) and pS6 (*n* = 8/8; Figures 3G–H’) in the FoxA2-overexpressing side, suggesting that mTOR signaling was inactivated by FoxA2. Conversely, co-expression of CA-mTOR with FoxA2 restored cell proliferation, as characterized by pHH3 expression compared with that in cells expressing FoxA2 alone (*n* = 7; Figures 3B–C’,M). The mTOR signal was also partly recovered in the co-electroporated neural tube (*n* = 6/7; Figures 3F,F’,I,I’). These results suggest that the negative effect of FoxA2 on mTOR signaling is rescued by CA-mTOR, and FoxA2 resides upstream of mTOR.

Actually the single electroporation of CA-mTOR upregulated pS6 and increased the number of positive cells for p-p70S6K and pHH3 (Supplementary Figure S1).

We asked if the changes of cell number were mediated by apoptosis, and performed a terminal deoxynucleotidyl transferase dUTP nick end labeling (TUNEL) assay. However, increasing positive signals were not detected after electroporation (*n* = 0/6 for control, *n* = 0/8 for FoxA2, *n* = 0/7 for FoxA2 + CA-mTOR; Supplementary Figures S2A–C’). We further considered the possibility that the apoptotic cells would have appeared at earlier stages, and carried out the same assay on the embryos of 24 hpt. However, no TUNEL-positive cells were found, either (*n* = 6 for all electroporations; Supplementary Figures S2D–F’), while the constitutively active Ptch (PtchΔ)-electroporated embryos showed positive signals, indicating the experimental system worked (Briscoe et al., 2001; Cayuso et al., 2006; Supplementary Figures S2G,G’). These results suggest that programmed cell death was not the main cause of the alterations in cell number.

We finally asked if the cell fate change were associated with mTOR signaling, and examined the expression of FoxA2 and Nkx2.2 in the electroporated samples. The results showed that most GFP-positive cells differentiated into the FoxA2-positive and the non-electroporated cells got Nkx2.2-positive, because the overexpressed FoxA2 induced the Shh expression that affects the surrounding area in a non-cell autonomous manner (Cho et al., 2014; Supplementary Figure S3).

We additionally carried out an *in situ* hybridization with the FP gene *F-spondin* (Klar et al., 1992; Burstyn-Cohen et al., 1999) probe. The results showed ectopic *F-spondin* expression in both neural tubes electroporated with FoxA2 alone (*n* = 8/8 for FoxA2; Figure 3K) or co-electroporated with FoxA2 and CA-mTOR (*n* = 6/7; Figure 3L), whereas such ectopic expression was not found in the control neural tube (*n* = 0/6; Figure 3J).

Taken together, these results indicated that FoxA2 negatively regulates cell proliferation by blocking the mTOR signal upstream of mTOR.

### RNF152 Is a Target Gene of FoxA2 and Is Expressed in the FP

The role of FoxA2 as a transcription factor led us to hypothesize that FoxA2 induces the expression of gene(s) that directly and negatively regulate mTOR signaling and cell proliferation. To identify such negative regulators of mTOR signaling expressed in the FP, we performed reverse transcription quantitative PCR (RT-qPCR) screening in chick neural explants.

**FIGURE 4:**
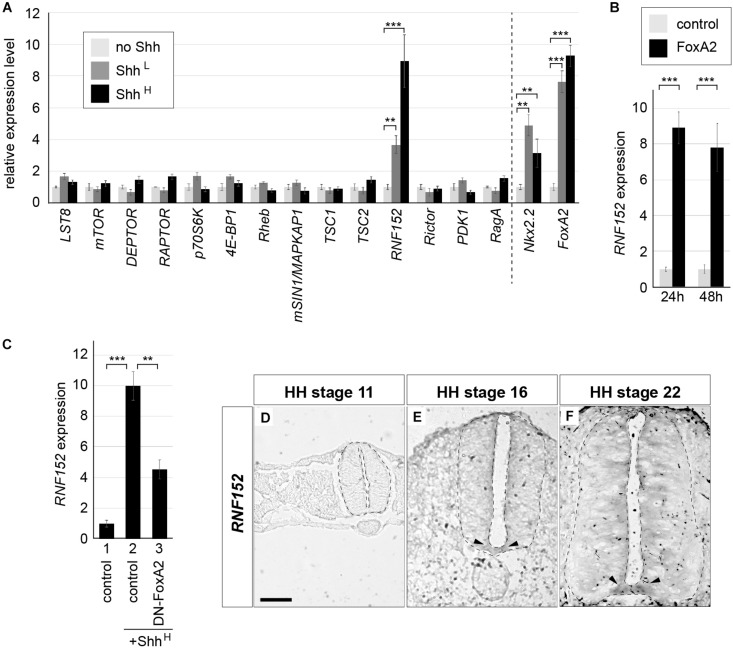
*RNF152* is one of target genes of FoxA2, and is expressed in the FP. **(A)** *RNF152* is a responsive gene for Shh. RT-qPCR analysis of genes related to the mTOR signal. Chick neural explants treated with control medium or in the presence of Shh_L_ or Shh_H_ for 48 h were analyzed using the indicated gene primers. **(B)** *RNF152* is a target gene of FoxA2. Explants electroporated with *pCIG* (control; gray) or *pCIG-FoxA2* (black) were cultured for 24 or 48 h and the expression of *RNF152* was analyzed by RT-qPCR. **(C)** FoxA2 is required for the *RNF152* expression. Explants electroporated with *pCIG* (control; lanes 1,2) or *pCIG-DN-FoxA2* (lane 3) were treated with control (lane 1) or Shh_H_-containing medium (lanes 2,3) for 48 h and the expression of *RNF152* was analyzed by RT-qPCR. **(D–F)** *RNF152* is expressed in the FP. Sections of the neural tube were analyzed by *in situ* hybridization with the *RNF152* probe at HH stage 11 **(D)**, 16 **(E)**, and 22 **(F)**. The FP expression is indicated by arrowheads **(E,F)**. Scale bar = 50 μm. ^∗∗^*p* < 0.01, ∗∗∗*p* < 0.001.

Neural explants treated with a high concentration of Shh (hereafter denoted as Shh_H_; see section “Materials and Methods” for the definition of “high concentration”) differentiate into the FP, whereas explants exposed to a low concentration of Shh (Shh_L_) tend to differentiate into motor neurons and V3 interneurons (Dessaud et al., 2010; Ribes et al., 2010; Sasai et al., 2014). RNA was extracted from explants exposed to Shh_L_ or Shh_H_ for 48 h, and gene expression levels were compared with those of explants without Shh by qPCR focusing on the components of mTORC1 (Laplante and Sabatini, 2009, 2012, 2013) (see Supplementary Table S1 for primer sequences). Proper upregulation of *Nkx2.2* and *FoxA2* expression by Shh treatment was confirmed (Dessaud et al., 2010; Sasai et al., 2014). The results showed that the expression of most of the genes was not affected by the presence or absence of Shh (4 trials each; Figure 4A). However, *RNF152*, which encodes an E3 ubiquitin ligase (Deng et al., 2015, 2019), was strongly induced in Shh_H_ explants, and a weaker induction was observed in Shh_L_ explants, suggesting that *RNF152* was expressed preferentially in the FP.

A previous study has shown that the regulatory region of the *RNF152* gene contains a FoxA2 binding region (Metzakopian et al., 2012). We therefore analyzed the relationship between FoxA2 and the expression of RNF152. For this purpose, we prepared explants electroporated with *FoxA2*, and compared gene expression with that of control-GFP electroporated explants at 24 and 48 hpt by RT-qPCR (3 trials each; Figure 4B). The *RNF152* transcription level was significantly higher in FoxA2-overexpressing explants than in GFP-electroporated explants, suggesting that *RNF152* is a target gene of FoxA2.

We further attempted to examine if the *RNF152* expression is dependent on FoxA2, and conducted another explant assay with an electroporation of the dominant-negative FoxA2 (DN-FoxA2) (Jacob et al., 2007). In the explants electroporated with the control plasmid treated with Shh_H_, the upregulation of the *RNF152* expression was found (Figure 4C, lanes 1,2). In contrast, the explants with DN-FoxA2 significantly reduced that expression (Figure 4C, lane 3), suggesting that the *RNF152* expression requires FoxA2.

To identify the spatial expression of *RNF152* in the neural tube, we performed an *in situ* hybridization analysis. Although the *RNF152* expression was not detected at HH stage 11 (Figure 4D), it was detected in the FP at HH stages 16 and 22, with a lower level of expression in the apical region of the neural tube (Figures 4E,F).

Taken together, *RNF152* is a downstream gene of FoxA2 and is a FP-specific regulator of the mTOR signal.

### RNF152 Negatively Regulates Cell Proliferation Through the mTOR Signaling Pathway

**FIGURE 5:**
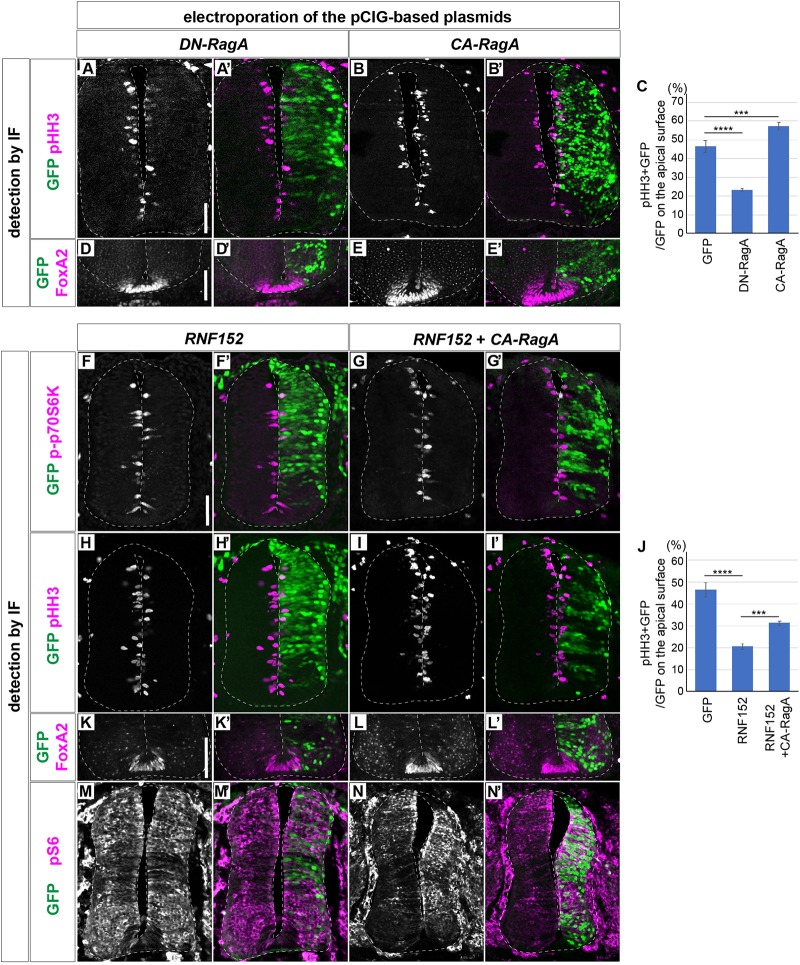
RNF152 negatively regulates the cell proliferation through the mTOR signaling pathway. **(A–E’)** Cell proliferation is regulated by the RagA activity. *pCIG-DN-RagA* **(A,A’,D,D’)** or *pCIG-CA-RagA* **(B,B’,E,E’)** was electroporated at HH stage 12 and phenotypes were analyzed at 48 hpt with pHH3 **(A–B’)**, FoxA2 **(D–E’)**, and GFP **(A’,B’,D’,E’)** antibodies. **(C)** Quantitative data for **(A–B’)**. The rates of the pHH3/GFP-double positive cells over the GFP-positive cells on the apical surface are presented. Scale bars in **(A)** for **(A–B’)** and in **(D)** for **(D–E’)** = 50 μm **(F–J)** RNF152 negatively regulates mTOR signaling and cell proliferation without altering the cell fate of the FP. *pCIG-RNF152* **(F,F’,H,H’,K,K’,M,M’)** or RNF152 together with CA-RagA **(G,G’,I,I’,L,L’,N,N’)** were electroporated at HH stage 12 and the p-p70S6K **(F–G’)**, pHH3 **(H–I’)**, FoxA2 **(K–L’)**, pS6 **(M–N’)**, and GFP **(F’,G’,H’,I’,K’,L’,M’,N’)** expression was analyzed by immunofluorescence at 48 hpt. Quantitative data for **(H–I’)** in **(J)**. The counting was performed as in Figures 3M, 5C. In **(C,J)**, the data of the control GFP electroporation is identical to that in Figure 3M. Scale bars in **(F)** for **(F–I’,M–N’)** and in **(K)** for **(K–L’)** = 50 μm. ∗∗∗*p* < 0.001, ∗∗∗∗*p* < 0.0001.

We next attempted to elucidate the function of RNF152 in the cell proliferation in the neural tube. The *RNF152* gene encodes an E3 ubiquitin ligase targeting the small GTPase RagA (Kim et al., 2008; Deng et al., 2015, 2019), and the GTP-bound active form of RagA positively regulates the mTOR signaling pathway (Shaw, 2008; Efeyan et al., 2014). The expression of the dominant-negative RagA (DN-RagA) blocks cell proliferation in the electroporated cells (Figures 5A,A’,C), whereas the constitutively active RagA (CA-RagA) activates it (Figures 5B,B’,C), as evaluated by pHH3 expression (Figure 5C) without changing the FP cell fate (Figures 5D–E’; *n* = 6 for DN-RagA and *n* = 7 for CA-RagA). RNF152 was therefore expected to act as a negative regulator of the mTOR signaling pathway by blocking RagA activity.

To prove this hypothesis, p-p70S6K expression was analyzed in cells overexpressing RNF152. The results showed that p-p70S6K was downregulated in response to RNF152 overexpression (*n* = 5/6; compare to the control GFP-electroporated embryos in Figures 3D,D’, 5F,F’), suggesting that RNF152 is a negative regulator of mTOR signaling. To examine the effect of RNF152 on cell proliferation in the neural tube, we analyzed the expression of pHH3 by immunofluorescence, which showed that the number of pHH3-positive cells was significantly lower in RNF152-overexpressing cells than in the cells electroporated with control GFP (*n* = 6/6; Figures 5H,H’; compare to Figures 3A,A’;Figure 5J for quantitative data). Therefore, RNF152 negatively regulates cell proliferation by blocking the mTOR signaling pathway.

We next examined whether the effect of RNF152 can be rescued by hyperactivation of RagA. For this purpose, we electroporated CA-RagA together with RNF152 (*n* = 7), and investigated the expression of p-p70S6K (Figures 5G,G’) and pHH3 (Figures 5I,I’). The results showed that the number of p-p70S6K- and pHH3-positive cells was significantly higher in cells co-electroporated with CA-RagA and RNF152 than in those overexpressing RNF152 alone (Figures 5F–J), whereas FoxA2 expression was unchanged (Figures 5K,K’,L,L’), suggesting that RNF152 *per se* is not involved in FP fate determination.

We next investigated if pS6 was affected by the electroporation of RNF152. The single electroporation of RNF152 did not significantly changed the localization of pS6, suggesting that the negative effect on the mTOR signal by RNF152 was compensated by other kinases in the intermediate region of the neural tube (Figures 5M,M’). However, there was a clear upregulation of pS6 by the coelectroporation of RNF152 and CA-RagA, suggesting that CA-RagA can activate S6 without perturbed by RNF152 (Figures 5N,N’).

In summary, RNF152 negatively regulates cell proliferation (i.e., pHH3) by blocking mTOR signaling (i.e., p-p70S6K) upstream of RagA.

### Blocking RNF152 Expression Leads to Aberrant Cell Division in the FP

To elucidate the function of RNF152 in mTOR signaling and FP cell proliferation, we designed a loss-of-function experiment to inhibit RNF152 expression by si-RNA. We electroporated *si-control* or *si-RNF152* in the ventral region of the neural tube together with the GFP-expressing plasmid at HH stage 10, and cultured the embryos for 48 h to reach HH stage 18.

**FIGURE 6:**
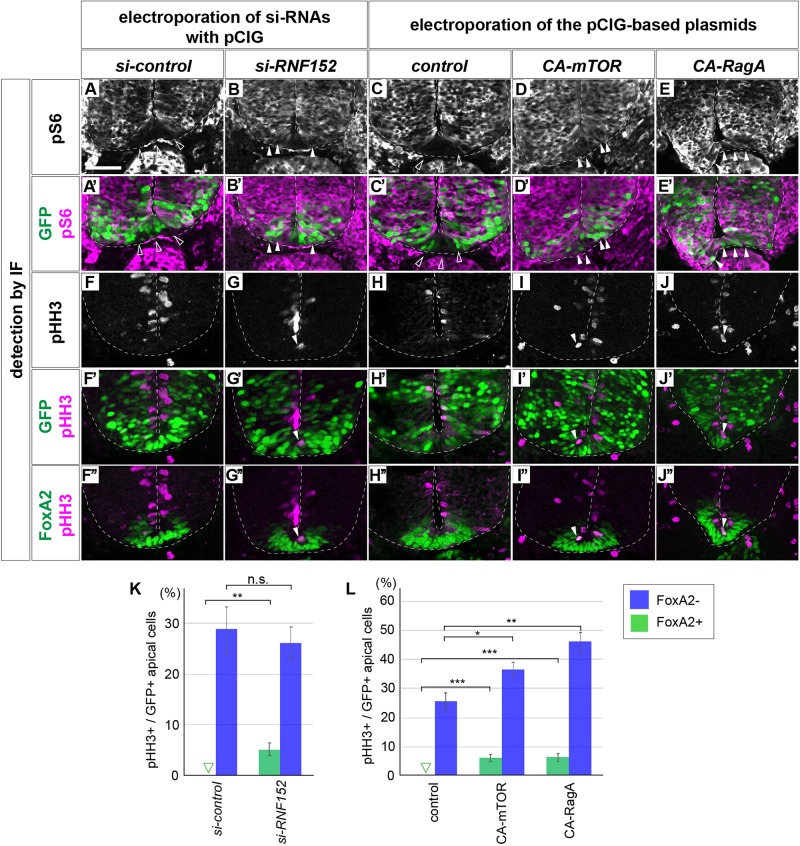
Blocking RNF152 expression or activation of mTOR signal leads to aberrant pS6 upregulation and cell division in the floor plate. **(A–B’,F–G”)** Knockdown of *RNF152* by *si-RNA* caused aberrant mTOR activation and the appearance of pHH3-positive cells. *si-control* **(A,A’,F,F’,F”)** or *si-RNF152* **(B,B’,G,G’,G”)** were electroporated in the FP at HH stage 10 and embryos were analyzed at 48 hpt with pS6 **(A–B’)**, pHH3 **(F–G”)**, FoxA2 **(F”,G”)**, and GFP **(A’,B’,F’,G’)** antibodies. **(C–E’,H–J”)** Activation of mTOR signal induces aberrant cell division. The plasmids of *control* pCIG **(C,C’,H,H’,H”)**, *CA-mTOR* **(D,D’,I,I’,I”)**, or *CA-RagA* **(E,E’,J,J’,J”)** was electroporated in the FP and analyzed with pS6 **(C–E’)**, pHH3 **(H–J”)**, FoxA2 **(H”–J”)**, and GFP antibodies **(C’,D’,E’,H’,I’,J’)**. The affected areas are indicated by filled arrowheads and outlined arrowheads. Scale bar = 50 μm. **(K,L)** Quantitative data for **(F,F’,G,G’)** in **(K)** and **(H,H’,I,I’,J,J’)** in **(L)**. The pHH3-positive cells in FoxA2/GFP double-positive cells (FoxA2+) and in FoxA2-negative (FoxA2–)/GFP-positive cells in the apical domain were counted. The outlined triangles indicate zero (0). ^∗^*p* < 0.05, ^∗∗^*p* < 0.01, ∗∗∗*p* < 0.001.

Although no ventral expansion of pS6 was observed in response to *si-control* electroporation (*n* = 0/8; Figures 6A,A’), *si-RNF152* induced aberrant pS6 expression in the FP (*n* = 6/7; Figures 6B,B’), suggesting that the mTOR signal can be reverted by inhibiting RNF152. Moreover, whereas pHH3 was not expressed in the *si-control*-electroporated neural tube (0/8; Figures 6F,F’,K), was detected in midline cells (6/7; Figures 6G,G’,K), The pHH3-positive cells co-expressed FoxA2 (*n* = 6/6; Figure 6G”; compared with *si-control* electroporated embryos in Figure 6F”), suggesting that the aberrant pHH3 expression was induced by the perturbation of RNF152 expression.

We confirmed that the activation of the mTOR signal induced the ectopic pHH3 expression in the FP region. We electroporated *control*, *CA-mTOR*, or *CA-RagA* expression plasmids in the ventral neural tube, and assessed the expression of pS6 and pHH3. As expected, the pS6 expression was detected in the FP region in response to *CA-mTOR* (6/6) and *CA-RagA* (*n* = 6/6) electroporation, whereas no expansion was observed in control (*n* = 0/8) electroporation (Figures 6C–E’; the double staining images of FoxA2 and pS6 in Supplementary Figure S4). Moreover, pHH3 expression, which was not detected in the FP upon the electroporation of the control plasmid, was detected in midline cells (Figures 6H,I,J,L). Moreover, the pHH3-positive cells co-expressed FoxA2 (*n* = 6/6 for CA-mTOR and 6/6 for CA-RagA; Figures 6I”,J”), suggesting that ectopic pHH3 expression did not change the FP cell fate (*n* = 0/8 for control, and 0/6 each for CA-mTOR and CA-RagA; Figures 6H”,I”,J”). Finally, the FoxA2 expression domain did not significantly change by electroporation of CA-mTOR or CA-RagA (8, 6, and 6 samples for control, CA-mTOR, and CA-RagA, respectively; Figures 6H’,I’,J’,L), suggesting that the FoxA2 expression was regulated at the upstream level or independently of the mTOR signal.

Taken together, RNF152 is essential for inhibiting cell proliferation, and blocking the function of RNF152 by *si-RNA* induced aberrant cell division in the FP. Moreover, the similar results were obtained by activating mTOR, which is consistent with a role of RNF152 in blocking mTOR.

## Discussion

### RNF152 Is a Negative Regulator of mTOR Signaling in Neural Tube Development

In the present study, we demonstrated that the mTOR signaling pathway is inactive in the FP region in the neural tube, and this inactivation corresponds to the low proliferation rate of FP cells. FoxA2 is a transcription factor that restricts the mTOR signal and cell proliferation (Figure 3), and this negative regulation is mediated by RNF152, a target gene of FoxA2 encoding an E3 ubiquitin ligase that targets the mTOR pathway component RagA.

Although Shh regulates not only pattern formation in the neural tube, but also cell proliferation and tissue growth, FP cells exposed to the highest level of Shh have a low proliferation rate (Kicheva et al., 2014). The present study elucidated the mechanism underlying this regulatory function.

RNF152, a lysosome-anchored E3 ubiquitin ligase (Zhang et al., 2010; Deng et al., 2019) containing RING-finger and transmembrane domains, was initially thought to induce apoptosis (Zhang et al., 2010). Further study showed that RNF152 ubiquitinates and targets the GDP-bound form of RagA for degradation, thereby negatively regulates mTOR signaling (Deng et al., 2015). Consistently, *RNF152* knockout cells exhibit hyperactivation of mTOR signaling (Deng et al., 2015). Moreover, a recent study proposed that RNF152 has an essential function in neurogenesis by regulating *NeuroD* expression (Kumar et al., 2017). Although these findings at the cell level suggest that RNF152 plays essential roles during the entire course of life including embryogenesis, mutant mice devoid of the *RNF152* gene are actually viable (Deng et al., 2015), suggesting the existence of a compensatory mechanism for RNF152 to ensure survival.

On the other hand, RagA, a substrate of RNF152, is essential for embryogenesis; genetic deletion of the *RagA* gene causes morphological and growth defects, and the embryos consequently die at embryonic day 10.5 (Efeyan et al., 2014). This suggests that other factors are also involved in the RagA activation. In addition, overexpression of RNF152 did not downregulate pS6 (Figures 5M,M’) despite a clear decrease of p-p70S6K (Figures 5F,F’), suggesting that p-p70S6K and pS6, two major readouts of mTOR signal (Fishwick et al., 2010; Biever et al., 2015), are regulated somewhat differently. Likewise, we showed that the localization of p-p70S6K and pS6 signals do not completely match; p-p70S6K was mostly detected at the apical surface along the D-V axis (Figures 2A–D), whereas pS6 was expressed at the transition zone between the progenitor and neuronal areas (Figures 2M–P). phospho-AKT (pAKT), which is a possible upstream regulator of the mTOR signal, was found at the apical domain of the neural tube and along the D-V axis (Supplementary Figures S5A,B), and later in the commissural axons (Supplementary Figure S5C). These findings suggest a complex transduction mechanism for the mTOR signal, and the activation of downstream components does not simply occur on a one-to-one basis. Therefore, although mTOR signal plays essential roles for the multiple aspects of the neural tube development, including cell proliferation (Figures 3, 5), differentiation (Fishwick et al., 2010), neural tube morphogenesis migration (Kobayashi et al., 2001; Torroba et al., 2018), and axon guidance (Supplementary Figure S5C), distinct intracellular factors are involved, and each critical function has to be analyzed and discussed separately (e.g., by using conditional knockout mice).

### mTOR and Other Signaling Pathways

**FIGURE 7:**
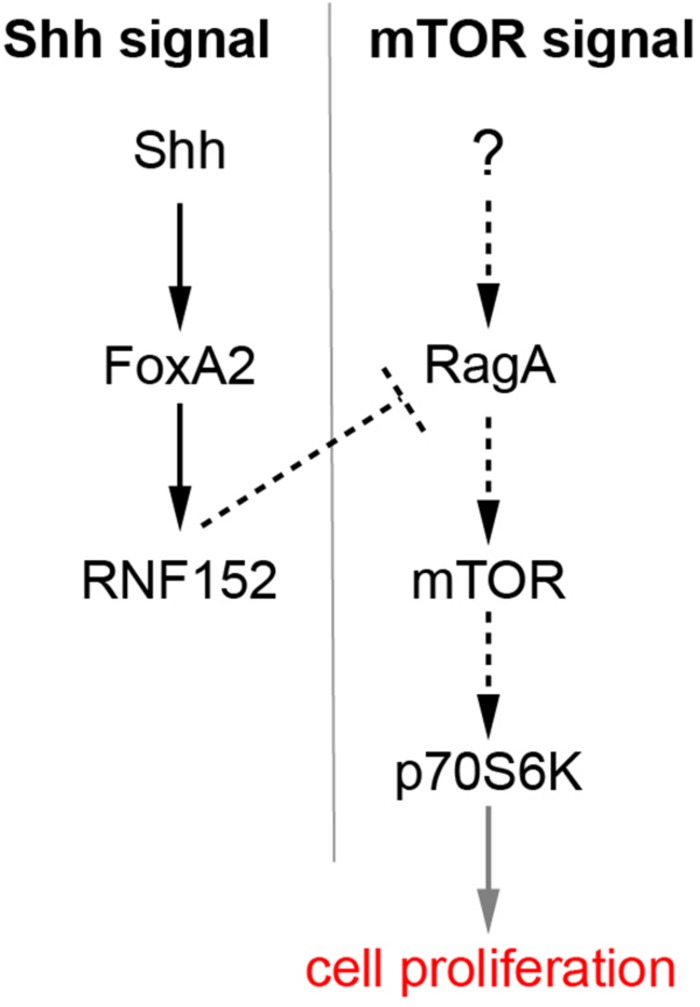
A regulatory loop composed of Shh, FoxA2, and RNF152 modulates FP cell proliferation. *FoxA2* expression is induced by Shh, whereas *RNF152* is a target gene of FoxA2. RNF152 blocks the cell proliferation through by negatively regulating mTOR signaling. Transcriptional regulation is indicated by solid arrows; activation, and inactivation with protein interactions or modifications are indicated by dotted arrows; the regulation of cell proliferation by the activation of p70S6K is apparently indirect, which is indicated by the gray arrow.

Figure 7 is a schematic of the regulation of cell proliferation in the FP. FoxA2, a target of Shh, and induces the expression of downstream target genes including *RNF152*. *RNF152* expression was not detectable at HH stage 11, immediately after the FoxA2 expression in the FP starts (Figure 4D), and the expression gradually increases during HH stage 11 to 16. By contrast, the mTOR signal is already inactive at HH stage 11. This apparent discrepancy can be explained either by the existence of additional negative regulators of mTOR signal and/or by the fact that the low level expression of the RNF152 is sufficient for the inhibition of the mTOR signal. We assume the *RNF152* induction by FoxA2 is direct, as suggested by the presence of a FoxA2-binding site in the enhancer region of the *RNF152* gene (Metzakopian et al., 2012).

The expression of RNF152 further inactivated the mTOR signaling pathway (Figure 5), thereby negatively regulating cell proliferation. In this sense, the present study linked the two signaling pathways mediated of Shh and mTOR. The inactivation of mTOR signal in the FP is consistent with the fact that mTOR signal is required for the neurogenesis (Fishwick et al., 2010), because neurogenesis does not occur in the trunk level of the FP (Placzek and Briscoe, 2005).

The upstream component of the mTOR pathway involved in cell proliferation remains unidentified (Figure 7). Insulin-like growth factors (IGFs) were introduced as mTOR activators in a number of cellular contexts (Laplante and Sabatini, 2012); therefore, IGF1/2 are potential candidates. IGF2 and the IGF1 receptor (IGF1R) are expressed in somites and in the dorsal part of the neural tube (Fishwick et al., 2010), and pS6 is found in the dorsal somite at early stages (Figure 2M) and in the dermomyotome at later stages (Figure 2N) in addition to its expression in the neural tube. Moreover, the pS6-positive regions in somites and dermomyotome are consistent with the findings of a previous report (Nie et al., 2018). In addition, IGF1R knockout mice exhibit impaired cell proliferation during brain development (Hu et al., 2012). Altogether, the upstream mTOR components localize to the right place at the right time. Further analysis will reveal the correlation between IGF and the mTOR signaling pathway.

The involvement of the mTOR signal in pattern formation should be analyzed in the future. The distribution of the mTOR active area along the D-V axis of the neural tube is not completely uniform, as shown by the localization of pS6 (Figures 2N–P), suggesting that the mTOR signal is somehow regulated by D-V patterning factor(s). As Wnt, BMP and Shh are essential for the D-V patterning, the interdependency of either of these factors with the mTOR signal is an intriguing question. For instance, the Wnt signaling pathway is inactivated in the *IGF1R* knockout mice (Hu et al., 2012). In addition, Shh signaling requires the mTOR signal (Riobo et al., 2006). Moreover, Shh and IGF signals cooperate with each other and promote cell proliferation in the brain (Rao et al., 2004) and osteoblast differentiation (Shi et al., 2015). Detailed analyses are necessary to elucidate the relationship between pattern formation and mTOR signaling, and thus the mechanisms underlying the determination of cell numbers in each neuronal region.

## Materials and Methods

### Ethics Statements

All animal experiments were carried out in accordance with the national and domestic legislations. All protocols used for the experiments on chick and mouse embryos were approved by the animal research review panel of Nara Institute of Science and Technology (approval numbers 1636 and 1810, respectively).

### Electroporation, Immunofluorescence, and *in situ* Hybridization

Chicken eggs were purchased from the Yamagishi Farm (Wakayama Prefecture, Japan), and developmental stages were evaluated according to the Hamburger and Hamilton criteria (Hamburger and Hamilton, 1992). Electroporation was performed with the ECM 830 (BTX) electroporator in the neural tube of embryos using pCIG-based expression plasmids, in which gene expression is induced by the chicken beta-actin promoter (Megason and McMahon, 2002). For ventral electroporation (Figure 6), electrodes were placed on and under the embryos. *pCIG-CA-mTOR* was generated by modifying the *pcDNA3-FLAG-mTOR-S2215Y* vector purchased from Addgene (# 69013), which was deposited by Dr. David Sabatini (Grabiner et al., 2014). While we normally got around 50% of survival rates in our electroporation experiments, the co-electroporation of *CA-mTOR* together with *FoxA2* (Figures 3C,F,I,L and Supplementary Figure S2C) seemed to be detrimental, and we got a lower survival rate (20–30%). Detailed information of the plasmids and *si-RNAs* used in this study is provided in Supplementary Table S2. Embryos were incubated in a 38°C incubator for the indicated times at constant humidity.

Embryos were fixed with cold 4% paraformaldehyde for 2 h, and then incubated with 15% sucrose/PBS solution overnight. Embryos were embedded in the OCT compound (Sakura) and sectioned at a thickness of 12 μm (Sakura Finetek, Japan).

Immunofluorescence and *in situ* hybridization were performed as described previously (Sasai et al., 2014). The antibodies used in this study are listed in Supplementary Table S2.

Timed pregnant mice were purchased from Japan SLC (Shizuoka Prefecture, Japan). Embryos were extracted and processed as described for chick embryos.

### Explants, RNA Extraction, and RT-qPCR

Intermediate neural explants comprise the uniform type of neural progenitor cells, which are sensitive to patterning factors and are a useful experimental model to recapitulate *in vivo* neural development (Dessaud et al., 2010; Sasai et al., 2014). For preparation, chick embryos were extracted from eggs at HH stage 9, and the intermediate region of the neural plate at the preneural tube level (Delfino-Machin et al., 2005) was excised. If necessary, expression plasmids were overexpressed before extracting the embryos (Figures 4B,C). Explants were embedded in a pH-adjusted collagen gel with DMEM. The culture medium consisted of DMEM/F-12 (Thermo Fisher Scientific), Mito + Serum Extender (Sigma), and penicillin/streptomycin/glutamine (Wako). Recombinant Shh was prepared in-house (Sasai et al., 2014; Kutejova et al., 2016). Shh_H_ was defined as the concentration at which the explants produced a dominant population of Nkx2.2-positive cells with a small subset of Olig2 cells at 24 h. SHH_L_ was defined as 1/4 of the concentration of Shh_H_, producing >70% Olig2-positive cells and a lower number of Nkx2.2-positive cells (Dessaud et al., 2007, 2010). At the late 48 h time point, Shh_H_ explants differentiated into the FP, whereas Shh_L_ induced motor neuron differentiation, as characterized by Islet1 expression (Ribes et al., 2010; Yatsuzuka et al., 2019).

For RT-qPCR, RNA was extracted using the NucleoSpin RNA extraction kit (Macherey-Nagel U0955), and cDNA was synthesized using the PrimeScript II cDNA synthesis kit (TaKaRa 6210). The qPCR reaction mixtures were prepared with SYBR FAST qPCR master mix (KAPA KR0389), and PCR amplification was quantified by LightCycler 96 (Roche). *GAPDH* was used as an internal control, and the quantification analyses were performed by the comparative Ct method.

### Image Acquisition and Statistical Analysis

Immunofluorescence and *in situ* hybridization images were captured with the LSM 710 confocal microscope and AxioCam digital camera (Carl Zeiss), and processed using Photoshop CC (Adobe). Images were integrated using Illustrator CC (Adobe). Statistical analyses were performed using Prism (GraphPad). Statistical data are presented as the mean ± SEM, and significance was set as follows: ^∗^*p* < 0.05; ^∗∗^*p* < 0.01; ^∗∗∗^*p* < 0.001; ^****^p < 0.0001; or n.s., not significant. The single comparison (Figures 1D, 4A,B, 6K,L and Supplementary Figure S1D) was tested by the two-tailed *t*-test, and multiple comparisons (Figures 3M, 4C, 5C,J) were performed by one-way analysis of variance (ANOVA) followed by Tukey’s *post hoc* test.

## Data Availability Statement

Data are available in the main text, figures, and the Supplementary Material.

## Ethics Statement

The animal study was reviewed and approved by the Nara Institute of Science and Technology.

## Author Contributions

NS conceived the project and drafted the manuscript. MK and NS performed the experiments and analyzed the data. MK edited the manuscript.

## Conflict of Interest

The authors declare that the research was conducted in the absence of any commercial or financial relationships that could be construed as a potential conflict of interest.

## Supplementary Material

FIGURE S1 Overexpression of *CA-mTOR* upregulates the positive cells for pS6, p-p70S6K and pHH3. **(A–C’)** *pCIG-CA-mTOR* was electroporated and analyzed as in Figure 3. The double-positive cells for indicated markers and GFP are indicated with white arrowheads. Scale bar = 50 μm. **(D)** Quantitative data for **(A,A’)**. ^∗^*p* < 0.05.

FIGURE S2 Programmed cell death is unlikely the main cause for the determination of the FoxA2-positive cell number. Plasmids expressing control GFP **(A,A’,D,D’)**, FoxA2 **(B,B’,E,E’)**, FoxA2 together with CA-mTOR **(C,C’,F,F’)**, or PtchΔ **(G,G’)** were electroporated into one side of the neural tube of HH stage 12 embryos, and the embryos were analyzed by a TUNEL assay at 48 hpt **(A–C’)**, or at 24 hpt **(D–G’)**. TUNEL-positive cells are presented with white **(A–G)** or magenta **(A’–G’)** signals, and the images combined with the GFP signals **(A’–G’)** are shown. Scale bars in **(A)** for **(A–C’)** and in **(D)** for **(D–G’)** = 50 μm.

FIGURE S3 Expression of Nkx2.2 and FoxA2 expression in the samples electroporated with the expression plasmids of *FoxA2* and *CA-mTOR*. The samples electroporated as in Figure 3 were analyzed with the Nkx2.2 **(A–C’,G,H,I)**, FoxA2 **(D–F’,G,H,I)**, and GFP **(A’–F’)** antibodies. Scale bar = 50 μm.

FIGURE S4 Blocking RNF152 expression or activation of mTOR signal leads to aberrant pS6 upregulation without affecting the FoxA2 expression in the floor plate. **(A_1_–B_5_)** *si-control* **(A_1_–A_5_)** or *si-RNF152* **(B_1_–B_5_)** were electroporated with *pCIG* (as the GFP tracer) in the FP at HH stage 10 and embryos were analyzed at 48 hpt with pS6 **(A_1_,A_3_,A_5_,B_1_,B_3_,B_5_)**, FoxA2 **(A_2_,A_4_,A_5_,B_2_,B_4_,B_5_)**, and GFP **(A_3_,A_4_,B_3_,B_4_)** antibodies. **(C_1_–E_5_)** The plasmids of *control* pCIG **(C_1_–C_5_)**, *CA-mTOR* **(D_1_–D_5_)**, or *CA-RagA* **(E_1_–E_5_)** was electroporated in the FP as in **(A_1_–B_5_)** and the samples were analyzed with pS6 **(C_1_,C_3_,C_5_,D_1_,D_3_,D_5_,E_1_,** **E_3_,E_5_)**, FoxA2 **(C_2_,C_4_,C_5_,D_2_,D_4_,D_5_,E_2_,E_4_,E_5_)**, and GFP antibodies **(C_3_,C_4_,D_3_,D_4_,E_3_,E_4_)**. The FP area is indicated by white arrowheads. Scale bar = 50 μm.

FIGURE S5 pAKT is localized at the apical domain and at commissural neurons. pAKT-positive cells are identified by immunofluorescence in chick neural tube sections HH stages 11 **(A)**, 16 **(B)**, and 22 **(C)**. Expression in the apical domain, in the commissural axons and in the dorsal interneuron are indicated by white arrowheads, white arrows and green arrowheads, respectively. Scale bar = 50 μm.

TABLE S1 Primers for quantitative PCR.

TABLE S2 Plasmids, siRNAs and antibodies used in this study (Tabancay et al., 2003; Li et al., 2004; Kim et al., 2008; Sato et al., 2008; Grabiner et al., 2014; Sasai et al., 2014; Yatsuzuka et al., 2019).
