# Supplementary figures and images for: Negative Regulation of mTOR Signaling Restricts Cell Proliferation in the Floor Plate
Source: Front Neurosci. 2019 Sep 25;13:1022. doi: 10.3389/fnins.2019.01022 (PMC6773814; doi:10.3389/fnins.2019.01022)

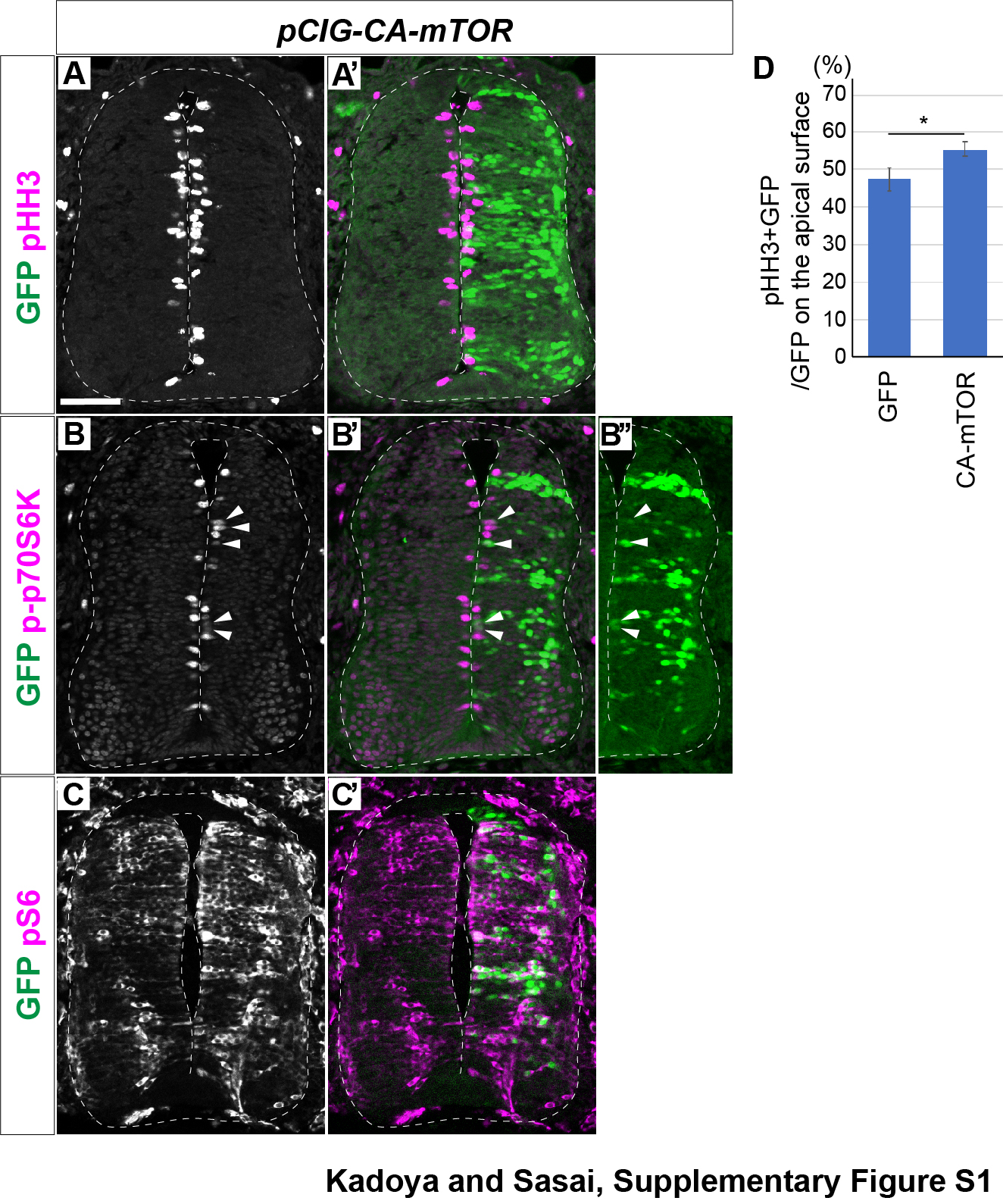

Supplement: FIGURE S1 — Overexpression of CA-mTOR upregulates the positive cells for pS6, p-p70S6K and pHH3. (A–C’) pCIG-CA-mTOR was electroporated and analyzed as in Figure 3. The double-positive cells for indicated markers and GFP are indicated with white arrowheads. Scale bar = 50 μm. (D) Quantitative data for (A,A’). ∗p < 0.05. [file Image_1.JPEG]

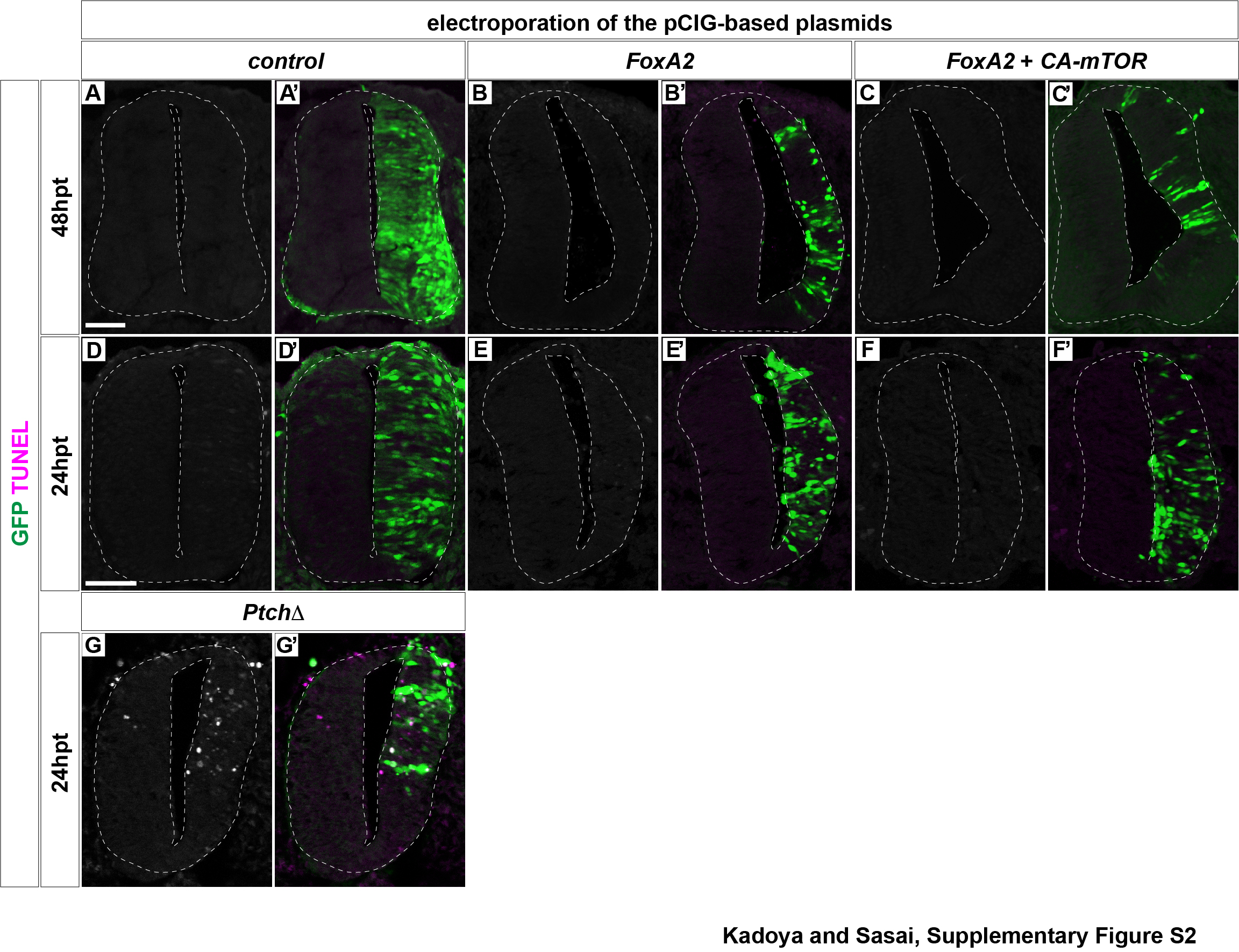

Supplement: FIGURE S2 — Programmed cell death is unlikely the main cause for the determination of the FoxA2-positive cell number. Plasmids expressing control GFP (A,A’,D,D’), FoxA2 (B,B’,E,E’), FoxA2 together with CA-mTOR (C,C’,F,F’), or PtchΔ (G,G’) were electroporated into one side of the neural tube of HH stage 12 embryos, and the embryos were analyzed by a TUNEL assay at 48 hpt (A–C’), or at 24 hpt (D–G’). TUNEL-positive cells are presented with white (A–G) or magenta (A’–G’) signals, and the images combined with the GFP signals (A’–G’) are shown. Scale bars in (A) for (A–C’) and in (D) for (D–G’) = 50 μm. [file Image_2.JPEG]

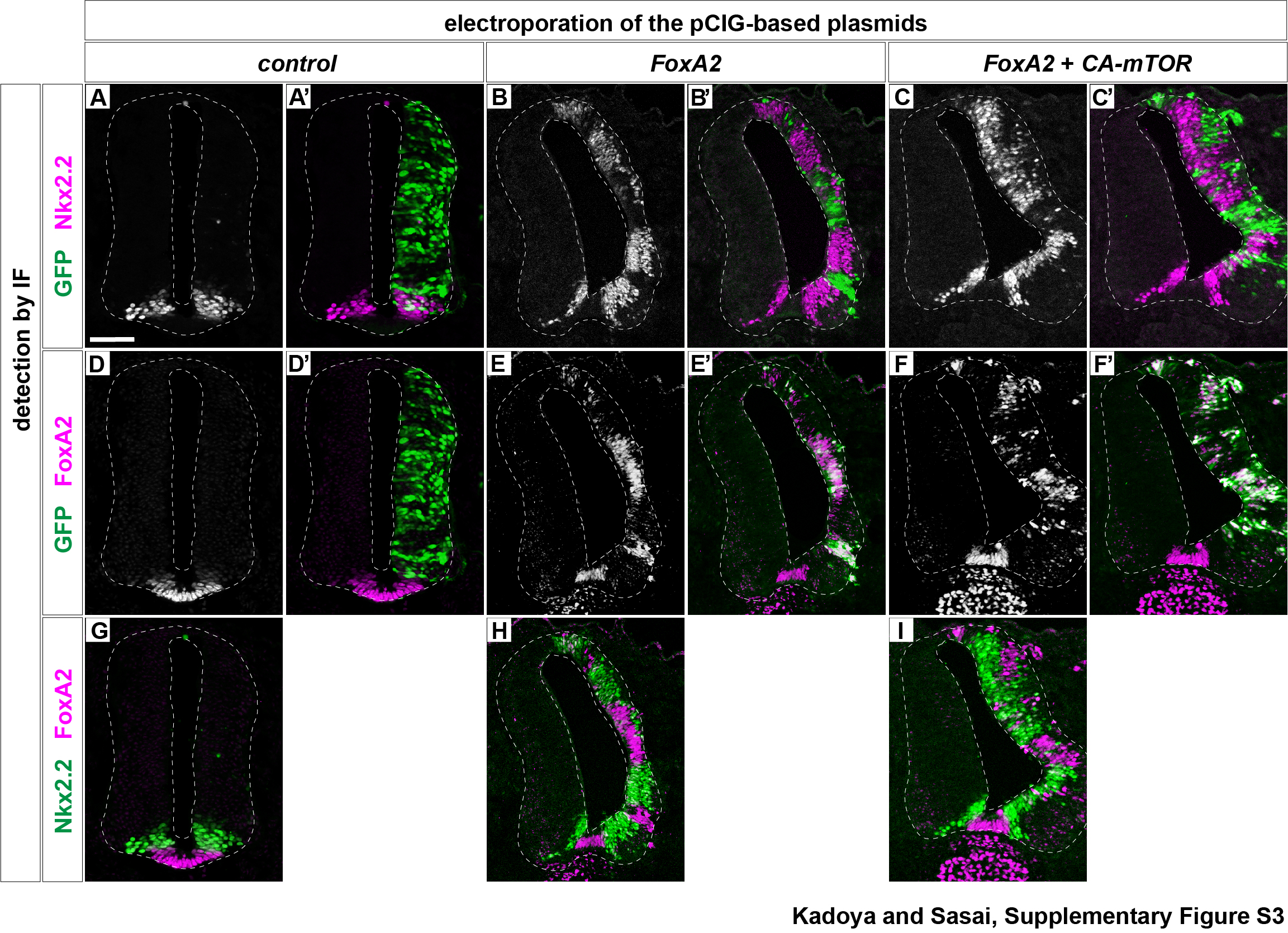

Supplement: FIGURE S3 — Expression of Nkx2.2 and FoxA2 expression in the samples electroporated with the expression plasmids of FoxA2 and CA-mTOR. The samples electroporated as in Figure 3 were analyzed with the Nkx2.2 (A–C’,G,H,I), FoxA2 (D–F’,G,H,I), and GFP (A’–F’) antibodies. Scale bar = 50 μm. [file Image_3.JPEG]

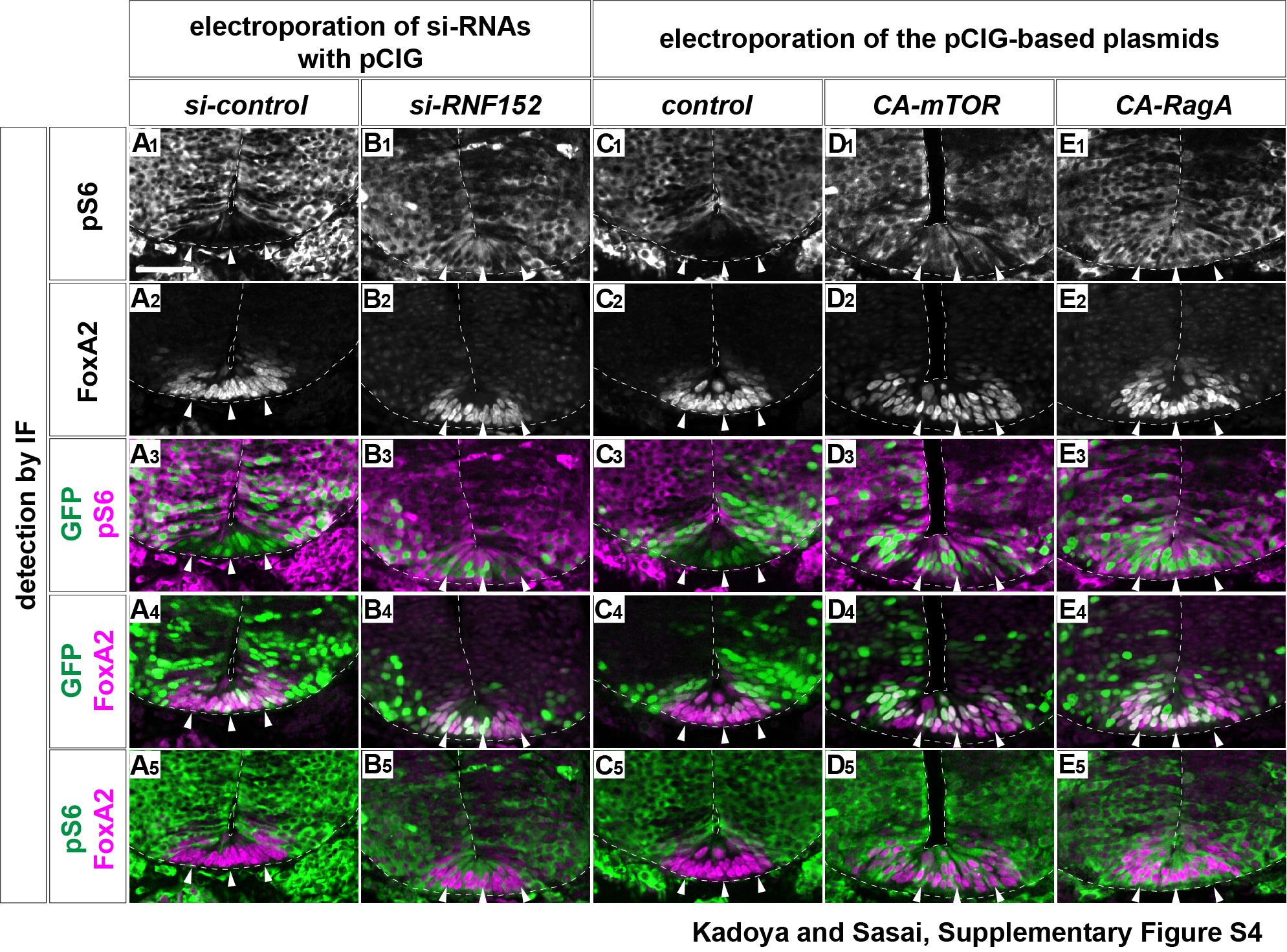

Supplement: FIGURE S4 — Blocking RNF152 expression or activation of mTOR signal leads to aberrant pS6 upregulation without affecting the FoxA2 expression in the floor plate. (A1–B5) si-control (A1–A5) or si-RNF152 (B1–B5) were electroporated with pCIG (as the GFP tracer) in the FP at HH stage 10 and embryos were analyzed at 48 hpt with pS6 (A1,A3,A5,B1,B3,B5), FoxA2 (A2,A4,A5,B2,B4,B5), and GFP (A3,A4,B3,B4) antibodies. (C1–E5) The plasmids of control pCIG (C1–C5), CA-mTOR (D1–D5), or CA-RagA (E1–E5) was electroporated in the FP as in (A1–B5) and the samples were analyzed with pS6 (C1,C3,C5,D1,D3,D5,E1, E3,E5), FoxA2 (C2,C4,C5,D2,D4,D5,E2,E4,E5), and GFP antibodies (C3,C4,D3,D4,E3,E4). The FP area is indicated by white arrowheads. Scale bar = 50 μm. [file Image_4.JPEG]

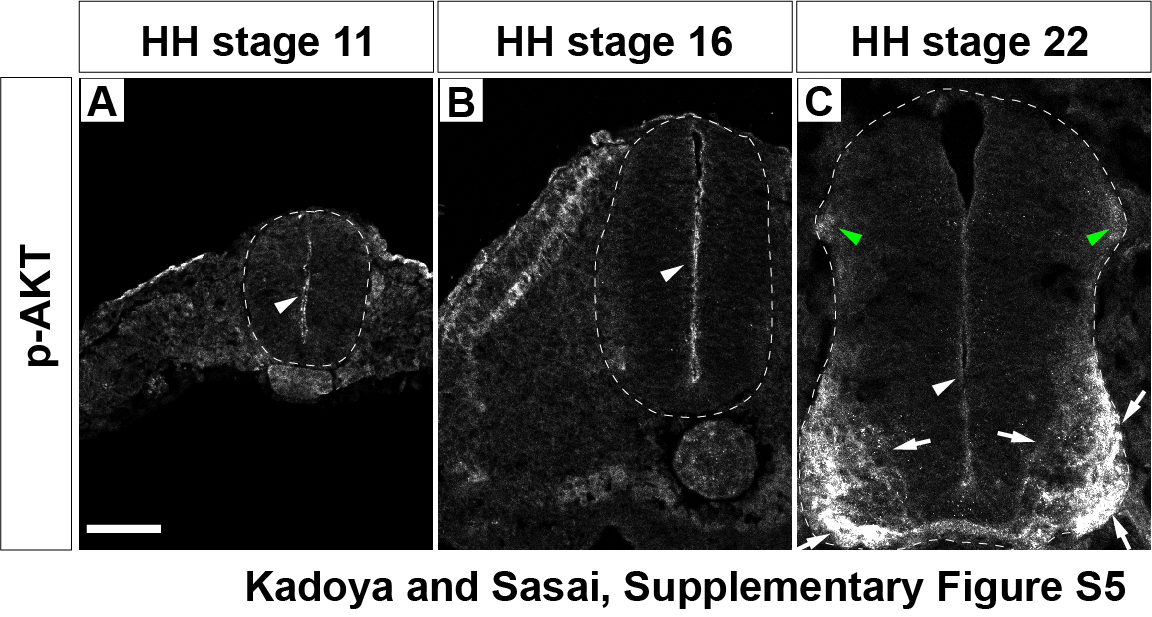

Supplement: FIGURE S5 — pAKT is localized at the apical domain and at commissural neurons. pAKT-positive cells are identified by immunofluorescence in chick neural tube sections HH stages 11 (A), 16 (B), and 22 (C). Expression in the apical domain, in the commissural axons and in the dorsal interneuron are indicated by white arrowheads, white arrows and green arrowheads, respectively. Scale bar = 50 μm. [file Image_5.JPEG]
